# Supplementary material for: Population genomics of Wolbachia and mtDNA in Drosophila simulans from California
Source: Sci Rep. 2017 Oct 17;7:13369. doi: 10.1038/s41598-017-13901-3 (PMC5645465; doi:10.1038/s41598-017-13901-3)
Supplement: Supplementary file 1 — Supplementary Information [file 41598_2017_13901_MOESM1_ESM.pdf]

Supplementary Information

Population genomics of *Wolbachia* and mtDNA in *Drosophila simulans* from California

Sarah Signor*

*Department of Molecular and Computational Biology, University of Southern California, Los Angeles, California USA

Key words: *Drosophila simulans*, population genetics, *Wolbachia*

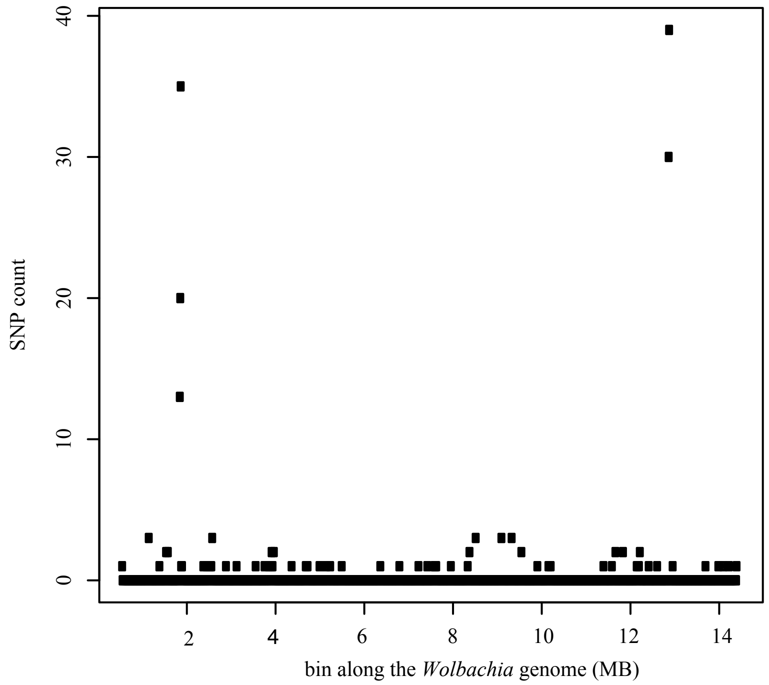

Supplementary Figure 1: SNP density in the *Wolbachia* genome. Two regions were excluded from the final set of SNPs due to unusual SNP density, shown here. These regions also coincided with regions of unusually high coverage, suggesting they are repetitive.

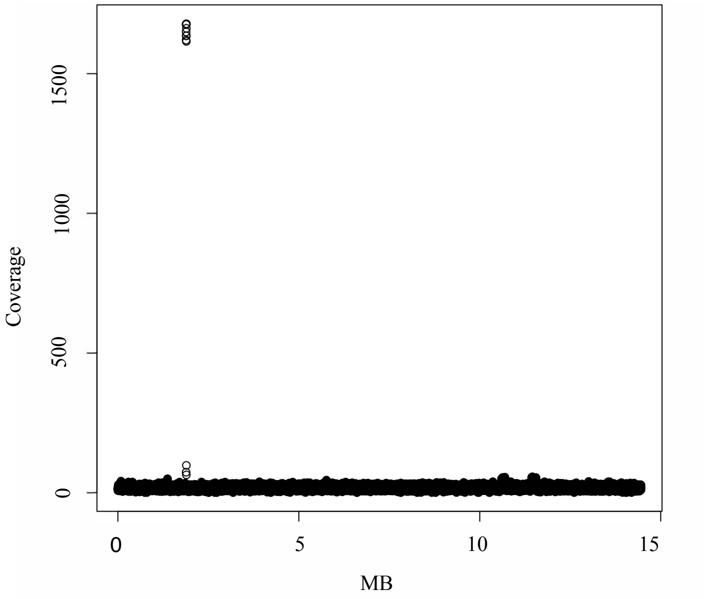

Supplementary Figure 2: An example of coverage across the *Wolbachia* genome, with a large outlier that was excluded from the final analysis. This region corresponds to one of the regions of unusually high SNP density, shown in Supp. Figure 1.

CHR	SNP	*p*-value
2R	5814103	1.47E-06
2R	5811053	3.37E-06
2R	13567097	5.61E-06
2R	13550916	6.41E-06
2R	13566886	6.72E-06
2R	13566065	6.87E-06
2R	13555805	7.01E-06
2R	13567885	7.06E-06
2R	13569038	7.27E-06
2R	13553992	7.29E-06
2R	13564712	7.29E-06
2R	13565153	7.42E-06
2R	13566442	7.65E-06
2R	13568212	7.70E-06
2R	13558515	7.97E-06
3L	2055556	8.24E-06
mtDNA	12354	3.18E-06

Supplementary Table 1: The full results of the association analysis. Note that while all significant results are included, 2R and 3L are the results of a multivariate analysis of *Wolbachia* and mtDNA titre while the significant hit for mtDNA was the result of a *Wolbachia* titre alone.
